# Genome sequencing in prenatal precision medicine of congenital malformations: Clinical experience from a single center

**DOI:** 10.1111/aogs.70381

**Published:** 2026-11-13

**Authors:** Eini Westenius, Peter Conner, Ellika Sahlin, Hafdis T. Helgadottir, Maria Pettersson, Meeli Sirotkina, Nikos Papadogiannakis, Anna Lindstrand, Erik Iwarsson

**Affiliations:** ^1^ Department of Molecular Medicine and Surgery Karolinska Institutet Stockholm Sweden; ^2^ Department of Clinical Genetics and Genomics Karolinska University Hospital Stockholm Sweden; ^3^ Department of Women's and Children's Health Karolinska Institutet Stockholm Sweden; ^4^ Center for Fetal Medicine Karolinska University Hospital Stockholm Sweden; ^5^ Department of Pathology Karolinska University Hospital Stockholm Sweden; ^6^ Department of Laboratory Medicine Karolinska Institutet Stockholm Sweden

**Keywords:** congenital abnormalities, fetal development, fetus, genetic diseases, genome sequencing, precision medicine, prenatal diagnosis

## Abstract

**Introduction:**

As a part of implementing genome sequencing in prenatal precision medicine at the department of Clinical Genetics and Genomics, Karolinska University Hospital, Stockholm, Sweden, we evaluated the utility of genome sequencing for prenatally detected malformations, including diagnostic yield, turnaround time and impact on current and future pregnancies.

**Material and Methods:**

This study comprised 218 fetuses with different malformations. One hundred and two prospectively collected samples were analyzed using clinical trio genome sequencing, and if non‐diagnostic, they underwent research analysis. In addition, 116 samples were collected retrospectively and included all fetuses referred for clinical trio genome sequencing during the study period. As part of the clinical routine at the time of the study, quantitative fluorescent‐polymerase chain reaction and chromosomal microarray had been performed before genome sequencing in 84.9% (185/218) of cases.

**Results:**

Clinical genome sequencing identified a molecular diagnosis in 22.5% (49/218) of the cases. Research analysis revealed two additional causative variants increasing the diagnostic yield to 23.4% (51/218). The phenotypes of the cases were divided into different groups with following diagnostic yields: 36.2% (21/58) in multisystem malformations, 30.8% (8/26) in skeletal malformations, 25.0% (14/56) in malformations in the central nervous system, 15.6% (5/32) in thoracic malformations, 11.5% (3/26) in abdominal malformations, and 0% (0/20) in other isolated anomalies. Additionally, four variants were considered potentially clinically relevant. A total of 39.2% (20/51) of the causative variants were related to a risk of recurrence. The turnaround time for clinical genome sequencing in ongoing pregnancies, when requested before gestational week 21 + 6 and prior to a parental decision on pregnancy continuation, was 12.5 ± 5 days (mean ± standard deviation, *n* = 40). Pregnancies with diagnostic results from genome sequencing were more likely to be terminated than those with normal results (*p* = 0.012, Fisher's exact test).

**Conclusions:**

Genome sequencing has an important role in prenatal precision medicine, since it can detect the underlying cause of prenatally detected malformations in a reasonable time. As causative chromosome aberrations were excluded in most of the cases, an even higher diagnostic yield can be expected if genome sequencing is offered as a first‐line test or after a normal rapid aneuploidy detection result.

## Graphical abstract

When a congenital malformation is detected prenatally, genome sequencing can help the clinicians when counseling parents on the management of pregnancy. An identified molecular diagnosis can also enable more accurate care of the pregnancy and the perinatal period, as well as provide more reproductive choices in future pregnancies.

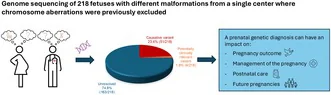

**Abbreviations**
CMA: chromosomal microarray analysis
CNV: copy number variant
ES: exome sequencing
GS: genome sequencing
QF‐PCR: quantitative fluorescence‐polymerase chain reaction
TAT: turnaround time
VUS: variant of uncertain significance

**Key message**

Genome sequencing is productive in the diagnosis of prenatally detected congenital malformations, as it detects different types of aberrations across the genome in a feasible turnaround time and as the result can have an impact on current and future pregnancies.

## 1 INTRODUCTION

Achieving a molecular diagnosis is one of the main goals in precision medicine, which is a medical approach that tailors the care and treatment of each individual patient based on their unique molecular and environmental data.^1^, ^2^, ^3^, ^4^ A prenatally achieved molecular diagnosis can help clinicians when counseling parents on the possible continuation and management of pregnancy. When parents following the diagnosis of a malformation decide to continue the pregnancy, a molecular diagnosis enables more accurate care of the pregnancy, including potential targeted treatments and individual management of labor and the perinatal period.^5^, ^6^, ^7^ Additionally, a molecular diagnosis can provide more reproductive choices in future pregnancies.

Congenital abnormalities occur in approximately 2.5%–3.5% of pregnancies.^8^, ^9^ Traditionally, genetic investigation of these conditions has included rapid aneuploidy detection and chromosomal microarray analysis (CMA) to detect aneuploidies and smaller copy number variants (CNVs). These genetic aberrations explain approximately 20%–25% of prenatally detected congenital abnormalities.^10^, ^11^, ^12^ However, the possibility of offering precision medicine has taken a significant leap forward after the discovery of high‐throughput sequencing technologies that can reveal the unique molecular data of each individual patient.^1^, ^2^, ^3^, ^4^ As genome sequencing (GS) can detect both coding and non‐coding single nucleotide variants and insertions and deletions, as well as different types of structural variants, short tandem repeats, and loss of heterozygosity in a single analysis, it offers an intriguing tool for prenatal precision diagnosis. Even if many ongoing initiatives for postnatal precision medicine use GS,^2^, ^3^, ^4^ there is a limited number of studies that have investigated the utility of GS in prenatal diagnosis. GS studies involving 96–295 fetuses with various malformations have previously presented a diagnostic yield of 15%–46%.^13^, ^14^, ^15^, ^16^

Since 2017, the Genomic Medicine Center Karolinska Rare Diseases has been bringing together healthcare and academia to implement precision diagnostics into the clinical world by enabling large‐scale GS to patients with rare diseases.^2^, ^4^ Within Genomic Medicine Center Karolinska Rare Diseases, our goal has been to implement publicly financed GS as a part of prenatal precision medicine. In this study, we investigate the clinical utility of trio GS in prenatal precision medicine of congenital malformations, including overall diagnostic yield, diagnostic yields in different phenotypic groups, turnaround time (TAT), and the possible clinical impact of GS on current and possible future pregnancies. We describe the clinical experience we have gathered and how we have systematically implemented GS, including a CNV analysis in GS data, as a part of prenatal precision medicine at our department.

## 2 MATERIAL AND METHODS

### 2.1 Study design

The prenatal cases were collected in two ways. One hundred and two prenatal cases were recruited prospectively between 30/03/2021 and 31/5/2024. The malformations of these cases were detected by prenatal ultrasound at the Center for Fetal Medicine, Karolinska University Hospital, Stockholm, Sweden. After the detection of a fetal malformation, the parents who chose invasive testing were invited to participate in the study involving also the potential research analysis. Additionally, parents who decided to terminate their pregnancy based on the findings in the prenatal ultrasound or whose pregnancy ended in perinatal death without invasive testing but who accepted postmortem examination of the fetus were invited to participate in the study. This participation required written consent by both parents. The results for the first 50 cases of this group analyzed with clinical trio GS have previously been published.^17^

To enable a more rigorous assessment of the clinical significance of GS in prenatal precision medicine, we retrospectively gathered information of all prenatal cases (*n* = 116), excluding the 102 prospectively collected cases already included in the study, with a malformation detected in prenatal ultrasound that were referred for clinical family‐based GS to the Department of Clinical Genetics and Genomics, Karolinska University Hospital between 11/4/2018 and 2/10/2025.

As one of the aims of this study was to establish the clinical indications for prenatal GS, we did not have set indications regarding which malformation cases should be offered prenatal GS during the study period. Instead, prenatal GS was offered based on clinical practice and consensus achieved in multidisciplinary discussions.

In cases (*n* = 18) where there were two fetuses with a prenatally detected congenital malformation in the same family, fetal samples were collected from both fetuses, and both were considered to be separate cases as the fetuses from the same family did not always have similar phenotypes. Additionally, in case #120, samples were collected from the affected fetus and a born affected sibling, but the affected sibling was not included in the study as a separate individual.

As quantitative fluorescence‐polymerase chain reaction (QF‐PCR) and CMA are the standard of care analyses in Sweden in the genetic investigation of prenatally detected congenital malformations, the prenatal cases were in most cases (*n* = 185) prescreened with QF‐PCR and CMA to exclude chromosome aberrations. In some cases, only QF‐PCR (*n* = 21) or CMA (*n* = 2) alone was performed before clinical GS. In 10 cases, no preceding analyses were performed before clinical GS. Cases with a causative variant detected by QF‐PCR or CMA were not included in the study.

The baseline characteristics of the 218 cases are presented in Table 1 and the more detailed clinical information in Table S1.

**TABLE 1:** Baseline characteristics.

Number of prenatal cases, *n*	218
Gestational age, median weeks+days (IQR)	19 + 3 (17 + 6–20 + 4)
Type of prenatal/postmortem sample, *n* (%)	
Amniocentesis	109 (50.0%)
Fetal tissue from postmortem examination	70 (32.1%)
Chorionic villus sampling	38 (17.4%)
Umbilical cord blood	1 (0.5%)
Sex chromosomes of the fetus, *n* (%)	
XY	130 (59.6%)
XX	85 (39.0%)
Sex chromosome aberration	3 (1.4%)
Primary clinical feature of the fetus, *n* (%)	
Multisystem	58 (26.6%)
Central nervous system	56 (25.7%)
Thorax	32 (14.7%)
Skeletal	26 (11.9%)
Abdominal	26 (11.9%)
Other isolated anomalies	20 (9.2%)
QF‐PCR and CMA prior to GS, *n* (%)	
No causative aberration detected in QF‐PCR and CMA	185 (84.9%)
No causative aberration detected in QF‐PCR	21 (9.6%)
No QF‐PCR or CMA performed	10 (4.6%)
No causative aberration detected in CMA	2 (0.9%)
GS approach, *n* (%)	
Trio	202 (92.7%)
Quad	15 (6.9%)
Duo	1 (0.5%)

Abbreviations: CMA, chromosomal microarray analysis; GS, genome sequencing; IQR, interquartile range; QF‐PCR, quantitative fluorescent‐polymerase chain reaction.

### 2.2 Research methods

The workflow of the study is presented in Figure 1. GS of DNA extracted from the fetal and parental samples was performed through an established collaboration with Clinical Genomics at Science for Life Laboratory (SciLifeLab, Solna, Sweden), which is a Swedish national center that provides high‐throughput bioscience through technical platforms, including massively parallel sequencing. The detailed analytical process has previously been described in Stranneheim et al.^2^ In brief, sequencing libraries were prepared using a PCR‐free paired‐end protocol: either Illumina TruSeq DNA PCR‐free for inputs with 1100 ng DNA or Lucigen NxSeq AmpFREE Low DNA for inputs with at least 200 ng but less than 1100 ng DNA. Short‐read sequencing was performed on the Illumina NovaSeq 6000 platform before 2023 and on the Illumina NovaSeq X platform from 2023, both with a 30X coverage. The bioinformatics analysis was conducted using the Mutation Identification Pipeline framework.^18^ In the Mutation Identification Pipeline framework, the FASTQ data was aligned to the human reference genome (hg19) following variant calling which included single nucleotide variants, insertions and deletions, structural variants, short tandem repeats, and copy number identification of the *SMN1* and *SMN2* genes. Identified variants were annotated, ranked and sent to our graphical user interface for massively parallel sequencing data and metadata called Scout.^2^

**FIGURE 1:**
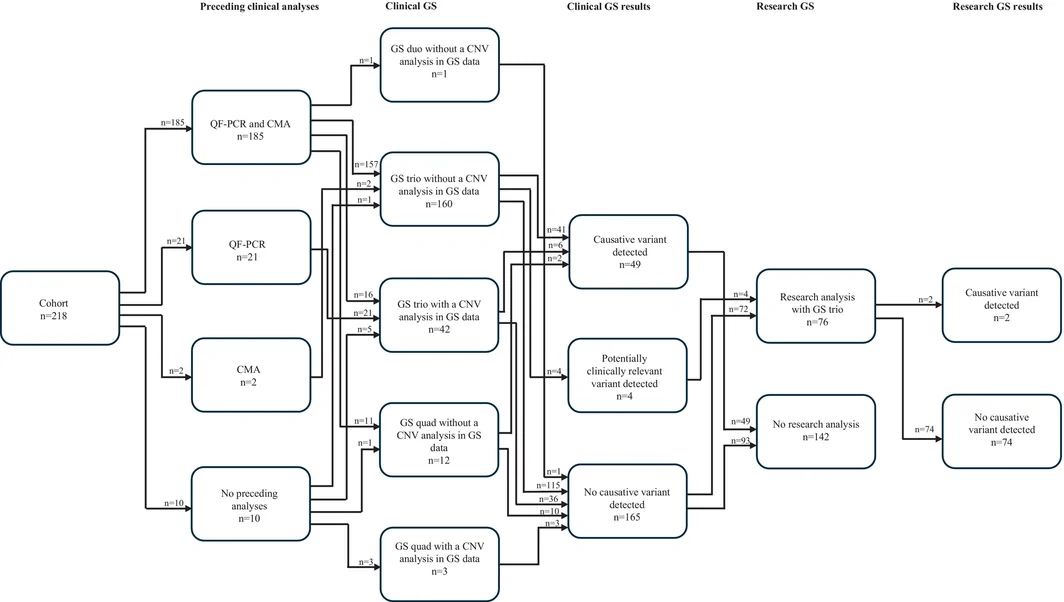
The workflow of the study. CNV, copy number variant; CMA, chromosomal microarray analysis; GS, genome sequencing; QF‐PCR, quantitative fluorescent‐polymerase chain reaction.

The clinical GS analyses were performed as fetal‐parental trio (*n* = 202), quad (*n* = 15) or duo (*n* = 1). An *in‐silico* gene panel based on all the morbid genes present in the Online Mendelian Inheritance in Man^19^ database at the given moment was used for filtering for disease‐associated genes in the clinical GS analysis. Variant classification was performed according to the guidelines provided by the American College of Medical Genetics and Genomics.^20^ Variants of uncertain significance (VUS's) considered as candidates based on the available phenotype were further discussed in a multidisciplinary team, including specialists in clinical genetics, fetal medicine and perinatal pathology. The prenatal phenotypes were based on the ultrasound findings in all cases. Additionally, in cases of pregnancy termination, perinatal death or death after the perinatal period, autopsy reports were reviewed if a postmortem examination had been performed and the autopsy report was available.

TAT was defined as days from the receipt of the sample at the Department of Clinical Genetics and Genomics to written report sent to the referring clinician.

If the clinical GS analysis of the 102 prospectively collected cases did not reveal an underlying genetic cause, the cases were directed to the trio GS research analysis (*n* = 76). In the research analysis, single nucleotide variants and insertions and deletions were analyzed in three filtrations: (1) the top 20 computably ranked exonic and predicted splicing variants with minor allele frequency <1%, (2) all exonic and predicted splicing autosomal dominant de novo variants, X‐linked variants (if a male fetus) and autosomal recessive variants (if known consanguinity) with minor allele frequency <0.01% and CADD^21^ > 30, and (3) all variants associated with 1–6 selected Human Phenotype Ontology terms.^22^ A list of the used Human Phenotype Ontology terms can be found in Table S1. Regarding structural variants, only the top 10 computably ranked exonic and predicted splicing variants were included in the research analysis.

In 45 cases, a whole‐genome analysis of large CNVs, corresponding to a CMA analysis, was performed using vcf2cytosure^23^ together with the GS. Vcf2cytosure enables visualization and interpretation of CNVs in the CytoSure Interpret Software (OGT, Oxford Gene Technology, Begbroke, United Kingdom) normally used in our laboratory to analyze CMA data.

### 2.3 Statistical analysis

The data are presented using mean and standard deviation, as well as median and interquartile range. Calculation of *p*‐values was performed via Fisher's exact test when applicable and *p* < 0.05 was considered to indicate statistical significance.

## 3 RESULTS

The clinical GS detected a causative variant in 22.5% (49/218) of the prenatal cases (Table S2). The trio GS research analysis of 76 fetal samples did not yield any high‐ranking research candidate genes but instead uncovered two additional causative variants (Table S3), increasing the total diagnostic yield to 23.4% (51/218) (Figure 2). Four additional variants were considered potentially clinically relevant even if they were assessed as VUS's according to the guidelines provided by American College of Medical Genetics and Genomics^20^ (Table S4). Overall, we identified a causative or potentially clinically relevant variant in 25.2% (55/218) of the cases.

**FIGURE 2:**
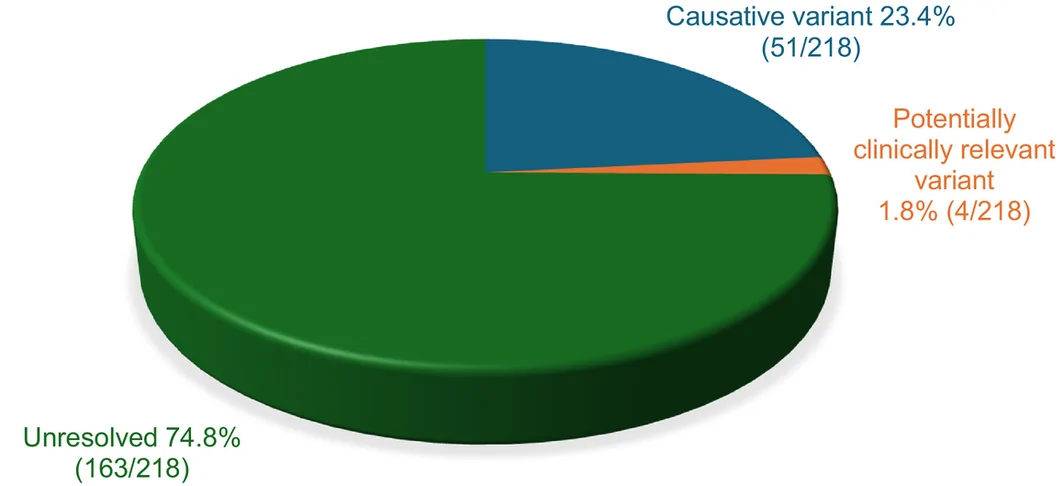
Diagnostic yield of the study including the research analyses.

Pathogenic incidental findings were detected and reported together with the other results from the GS analysis to the families in 17 cases (Table S5). Four of these cases came from two families (#122 and #123 as well as #208 and #209). The incidental findings were considered to not explain the phenotype of the fetus but were reported since they were reported earlier in a preceding QF‐PCR/CMA (cases #36, 37, #69, #129, #174, #208, and #209) or since they were deemed medically actionable (cases #48, #61, #122, #123, #145, #179, #182, #187, #192, and #193).

In 39.9% (87/218) of the cases, the phenotype that was available for the GS was purely based on the prenatal ultrasound findings. In this group, 23.0% (20/87) of the fetuses carried a causative variant and 2.3% (2/87) had a potentially clinically relevant variant. In 60.1% (131/218) of the cases, the phenotype of the fetus that was available for the GS was based on both the ultrasound findings and a postmortem examination. In this group, 23.7% (31/131) of the fetuses carried a causative variant and 1.5% (2/131) had a potentially clinically relevant variant.

The cases were categorized into six distinct phenotypic groups based on the primary affected organ system (Table 1). Causative variants were found most frequently in the phenotypic groups of multisystem malformations, with a diagnostic yield of 36.2% (21/58), skeletal malformations, 30.8% (8/26), and malformations in the central nervous system, 25.0% (14/56). Causative variants were detected to a lesser extent in association with thoracic malformations, 15.6% (5/32), and abdominal malformations, 11.5% (3/26). No causative variants were detected in fetuses with other isolated anomalies. The group‐specific diagnostic yields are presented in Figure 3.

**FIGURE 3:**
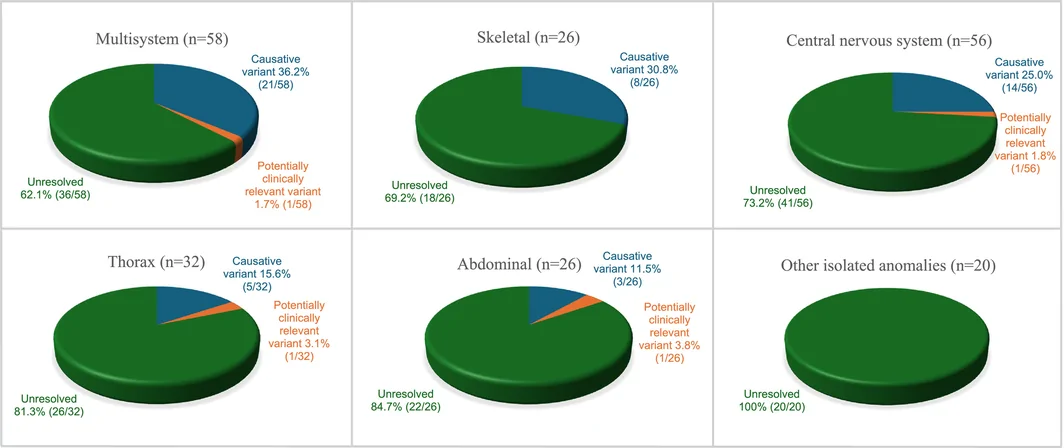
Diagnostic yields in the different phenotypic groups including the research analyses.

The TAT of the clinical GS varied depending on the pregnancy status (ongoing vs planned termination) and the gestational age. Eighty‐four out of 218 (38.5%) of the pregnancies were ongoing during the start of the clinical GS. Forty out of 84 (47.6%) of these pregnancies had a gestational age of less than 21 + 6 pregnancy weeks (which is the limit for late pregnancy termination in Sweden), and these parents had not decided on the continuation of the pregnancy before the GS was initiated. In 4/40 of these pregnancies, the parents decided to terminate the pregnancy before the GS result was available, and 1/40 of these pregnancies resulted in miscarriage before the GS result was available. However, the TAT of the ongoing GS was not affected by these pregnancy outcomes. All in all, the TAT of the clinical GS in these 40 pregnancies was 12.5 ± 5 days (mean ± standard deviation, *n* = 40). In the other ongoing pregnancies, the clinical GS was performed so that the results were reported at latest before childbirth. The individual TATs are presented in Table S1.

We investigated the clinical impact of prenatal GS on pregnancy outcomes and on the counseling of recurrence risk. We were also able to gather information regarding the postnatal impact of a prenatally detected genetic disorder in cases where the pregnancy resulted in a live birth.

All the pregnancy outcomes and the possible impact of the clinical GS analysis on the pregnancy outcome are presented in Figure 4. There was a significant relation between the outcome of the pregnancy and the results from the clinical GS analysis in ongoing pregnancies (*p* = 0.012, Fisher's exact test). When the GS was requested before gestational week 21 + 6 and the results from the GS analysis were delivered prior to a parental decision on pregnancy continuation, 72.7% (8/11) of the parents receiving a molecular diagnosis for the prenatal condition terminated their pregnancy, whereas only 25.0% (6/24) of the parents receiving non‐diagnostic results from prenatal GS terminated their pregnancy (Table 2).

**FIGURE 4:**
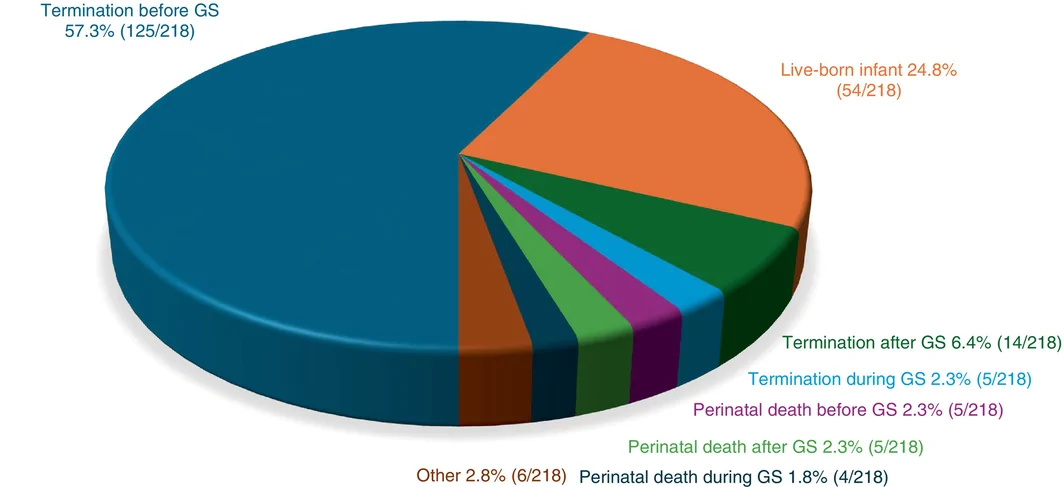
Pregnancy outcomes. The prepositions of time refer to a pregnancy outcome that happened either before the GS was initiated, during the ongoing GS analysis or after the GS was performed and results from it were available. The category “other” includes the following pregnancy outcomes: Miscarriage before GS 1.4% (3/218), miscarriage during GS 0.5% (1/218), death after perinatal period before GS 0.5% (1/218) and death after perinatal period after GS 0.5% (1/218). Perinatal period is defined as a time period from 22 completed weeks of gestation to 7 completed days after birth. Miscarriage is defined as a spontaneous loss of pregnancy before 22 completed weeks of gestation. GS, genome sequencing.

**TABLE 2:** Impact of genome sequencing on pregnancy outcomes.

	Number of cases	Number of cases that chose to terminate their pregnancy	Number of cases that chose to continue their pregnancy
Diagnosed	11	8	3
Undiagnosed	24	6	18
Overall	35	14	21

*Note*: In the included 35 cases, genome sequencing was requested before gestational week 21 + 6 (which is the limit for late pregnancy termination in Sweden) and the results from the genome sequencing analysis were delivered prior to a parental decision on pregnancy continuation.

There were six cases where the prenatally detected genetic diagnosis had a clinical impact on not only the parental decision to continue pregnancy, but also on the decision on the management of pregnancy and/or postnatal care. In case #5 with a causative variant in *PDHA1* causing pyruvate dehydrogenase E1‐alpha deficiency, a specific diet and medication were planned before birth and started directly after birth for this potentially lethal condition. In case #86, where a causative variant in *PTPN11* associated with Noonan syndrome 1 was detected, the prenatally verified molecular diagnosis enabled more targeted surveillance of the pregnancy, including planning of the delivery, as well as individualized postnatal care, including possible prophylactic treatment to prevent ventricular tachycardia, and follow‐up of the born child. In case #214 with a causative variant in *SMARCC1* associated with congenital hydrocephalus, the prenatal diagnosis helped in planning the delivery method and provided individualized management of the hydrocephalus without need for a shunt. In cases #6 with a causative variant in *COL1A2* associated with osteogenesis imperfecta, #169 with a causative variant in *CEP55* associated with MARCH syndrome, and #177 with a causative variant in *ABAT* associated with GABA‐transaminase deficiency, the conditions were assessed to be lethal postnatally prompting a modified obstetrical plan with vaginal birth without fetal monitoring followed by comfort care of the child. In case #21 with a causative variant in *ACTA1* associated with Nemaline myopathy 3, the child was stillborn. In case #66 with a causative variant in *CDH7* causing CHARGE syndrome, the child was born 5 days after the GS was initiated, and the results from GS confirmed CHARGE syndrome. The live‐born infants were not followed up post‐delivery in the present study.

A total of 39.2% (20/51) of the detected causative variants were related to a risk of recurrence in future pregnancies (Figure 5). However, two cases (#115 and #116) were from the same family. A total of 57.9% (11/19) of the parents with a recurrence risk in future pregnancies have already started the process of preimplantation genetic testing to try to achieve a normal future pregnancy. Of the cases with reported incidental findings, three more families have initiated preimplantation genetic testing. The number of parents going through invasive prenatal testing in future pregnancies was not followed in this study.

**FIGURE 5:**
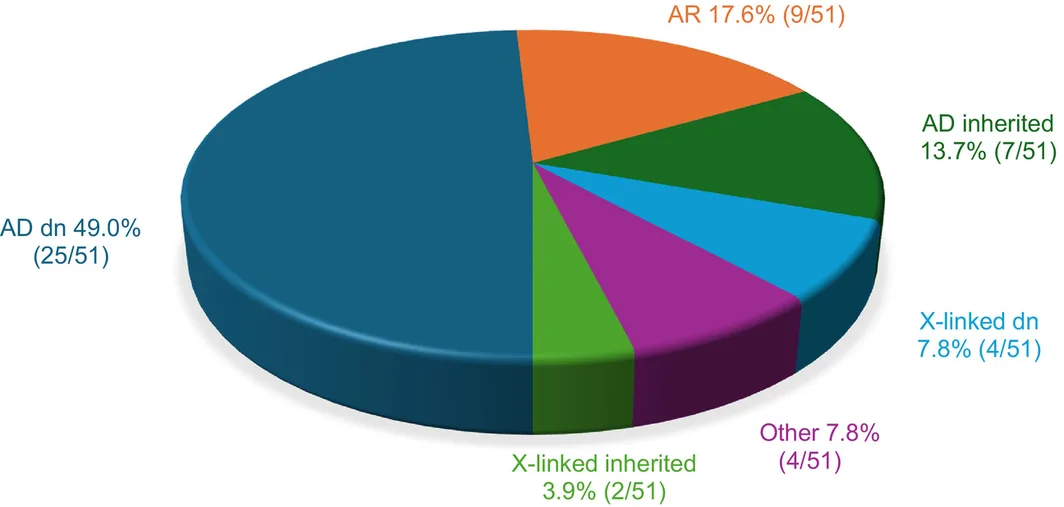
Inheritance pattern of the causative variants. The category “other” consists of maternal uniparental isodisomy of chromosome 1 (case #72), a mitochondrial variant with homoplasmy both in fetus and mother (case #180), maternal uniparental heterodisomy of chromosome 14 (case #194), and an unbalanced translocation due to a balanced reciprocal translocation in the mother (case #204). AD, autosomal dominant; AR, autosomal recessive; Dn, de novo.

## 4 DISCUSSION

This study was conducted as a part of a prenatal precision medicine initiative to implement GS for investigation of prenatally detected congenital malformations within the publicly financed regional health care system. In this study, a clinical trio GS provided a genetic diagnosis in 22.5% (49/218) of the cases with prenatally detected congenital malformations. Most of the cases were prescreened for trisomies and CNVs as QF‐PCR and CMA are the main standards of care analyses in Sweden when investigating prenatally detected congenital malformations. Research analysis using GS data on 76 cases detected two additional causative variants increasing the diagnostic yield to 23.4% (51/218). Moreover, four additional cases harbored variants considered potentially clinically relevant, resulting in a total of 25.2% (55/218) of cases with a causative or potentially clinically relevant variant (Figure 2). This diagnostic yield is in the range of the diagnostic yields (15%–46%) of the four previously published GS studies involving 96–295 fetuses with various malformations.^13^, ^14^, ^15^, ^16^

High‐throughput sequencing technologies are now widely adopted to detect the underlying molecular diagnosis of a patient, which is essential for precision medicine to be successful.^1^, ^2^, ^3^, ^4^ Both exome sequencing (ES), especially together with a CMA analysis, and GS have proven to be important tools in the prenatal diagnosis of congenital malformations by increasing the diagnostic yield compared to rapid aneuploidy detection and/or CMA alone.^13^, ^14^, ^15^, ^16^, ^24^, ^25^ As GS enables the identification of a broad range of genetic aberrations across the whole genome, including variants in non‐coding regions of the genome and complex structural variants, it has the potential to reach a higher diagnostic yield using a single method than ES. Since fewer studies have assessed the use of prenatal GS than prenatal ES in prenatally detected congenital malformations, it is currently difficult to compare the diagnostic performance of the two methods. A recent systematic review compared eight cohorts with a total of 755 prenatal cases with congenital malformations and stated that the overall diagnostic yield of GS over CMA was 16%–23%.^25^ Only two of the included studies compared the diagnostic yield of GS over ES and found that GS had an incremental yield of only 1%–2% over ES.^25^ The reason why GS has not yet reached a distinctly higher diagnostic yield than ES in the prenatal setting can be due to the complexity of the interpretation of the variants detected by GS but missed by ES. Interpreting for example, variants in non‐coding regions of the genome usually requires experimental and computational methods, which can be challenging to perform in the time pressure of an ongoing pregnancy. As GS at present focuses on variants located in coding regions and in intronic regions close to exon‐intron junctions, as well as on a limited number of non‐coding areas known to contain potentially clinically relevant variants, most of the variants identified with GS would most likely be detectable with a combined CMA and ES approach. Nonetheless, the diagnostic yield of GS is likely to increase in future when new knowledge regarding variants in non‐coding regions and complex structural variants emerges.

On the given moment of our study, CMA was still a standard analysis in the genetic investigation of prenatally detected congenital malformations at our department. Thus, causative chromosome aberrations were excluded prior to GS in most of the cases in our study. Hence, our study cannot make a full comparison between GS and a combination of ES and CMA. Most of the detected variants in our study would have also been detectable with ES. However, our study still demonstrates some of the opportunities that GS can offer compared to ES and/or CMA. We detected a causative variant in mitochondrial genes *MT‐ATP6* and *MT‐ATP8* in case #180, which could have been missed with ES as mitochondrial variants are detected with ES only if off‐target reads are added to the analysis. In two other cases, causative chromosome aberrations were detected first with clinical GS analysis. In case #194, a maternal uniparental heterodisomy of chromosome 14 was detected with clinical GS but not with our array‐CGH analysis. In case #204, that had not gone through a preceding CMA analysis, an unbalanced translocation was detected with our clinical GS analysis. These cases are good examples of the potential of GS as a first‐line test without a preceding CMA. In fact, our accumulated experience of CNV analysis based on GS data,^2^, ^4^ has enabled us to replace our CMA analysis with a GS based CNV analysis in prenatal cases undergoing GS. Given that roughly 20%–25% of fetal malformations are caused by chromosomal aberrations,^10^, ^11^, ^12^ an even larger diagnostic yield than 23.4% can be expected for these cases if GS is offered as a first‐line test or after a normal QF‐PCR result. However, more studies are needed to confirm this.

Detecting the potential underlying genetic cause of a congenital malformation depends not only on the method used but also on the prenatal phenotype and its severity. The unique challenge in prenatal genetic diagnosis is the prenatal phenotype, which often is not as complete as a postnatal phenotype. Additionally, the number of prenatal cases with the same or similar phenotype is lower than postnatal cases with a similar phenotype. These factors make the variant interpretation more challenging and hence, possibly result in a lower diagnostic yield of a prenatal genetic analysis compared to a postnatal analysis. In our study, we included a variety of malformations, from single organ anomalies to severe symptoms from multiple organs, to investigate the diagnostic yield of GS in different phenotypic groups. The research analysis also allowed us to include cases with a milder phenotype than those we would currently offer clinical prenatal GS to. With this approach, we might have decreased the diagnostic yield of the study as a genetic diagnosis is more likely to explain multiorgan anomalies than single anomalies, a fact demonstrated by us and other studies.^13^, ^14^, ^16^, ^24^, ^25^ The diagnostic yield of a method does not only depend on the number of detected malformations but also on the specific phenotypic groups (Figure 3). Based on this knowledge, we now offer trio GS using an Online Mendelian Inheritance in Man^19^ gene panel (5077 genes per date 2026‐07‐09) and a GS based CNV analysis for specific prenatal indications (Table 3) as a clinical routine.

**TABLE 3:** Current indications for clinical prenatal trio genome sequencing.

Arthrogryposis
Bilateral jugular lymphatic cysts and nuchal translucency ≥3.5 mm
Central nervous system malformation (excluding spina bifida)
Complex heart defect
Diaphragmatic hernia
Enlarged echogenic or cystic kidneys
Hydrops fetalis
Nuchal translucency ≥6 mm
Multiple malformations (≥ 3 organ systems)
Omphalocele
Severe growth restriction (≤ −33%) without signs of placental insufficiency
Skeletal dysplasia

*Note*: Trio genome sequencing with genome sequencing based copy number variation analysis is offered to prenatal cases with at least one of the above‐mentioned symptoms detected by prenatal ultrasound or postmortem examination.

Even if a possible postmortem examination following an intrauterine fetal death or a termination of pregnancy can add to the phenotype, there was no clear difference between the diagnostic yields in the group of fetuses with purely prenatal phenotype (23.0%, 20/87) and the group of fetuses with additional phenotypic description from a postmortem examination (23.7%, 31/131) at the time of the GS in our study.

The possibility of an incomplete prenatal phenotype, the lack of reported prenatal cases with rare genetic disorders and the lack of interpretational knowledge of for example, variants in non‐coding regions and complex structural variants, do not only decrease the diagnostic yield of GS but also make the likelihood of detecting a VUS higher. The decision to report VUS's during an ongoing pregnancy can be challenging and should be considered carefully. In cases #26 and #51 with a reported VUS in *FLVCR2* and *SLC9A3* respectively, the genotype–phenotype correlation was assessed to be so strong that the variants were considered clinically causative even if they were assessed as VUS's according to the American College of Medical Genetics and Genomics guidelines.^20^ The two other VUS's (cases #25 and #102) were reported because further investigation in the family was considered to be helpful in the variant interpretation. However, in case #25 with a fetus with a cardiovascular anomaly with dextrocardia but without situs inversus, an echocardiogram of the mother showed normal results. This meant that we could not tell whether the variant in *NODAL*, detected both in fetus and mother, was causative or not, even if potential incomplete penetrance of variants in *NODAL* has been described at least once in the literature.^26^ In case #102 with a male hydropic fetus, the same *IKBKG*‐variant was found in another affected fetus of the parents but because the history and clinical investigations of the carrier mother could not show any sign of incontinentia pigmenti, the variant in *IKBKG* was also considered as a VUS.

To explore what may remain undetected in the clinical GS analysis and to search for novel disease‐causing variants and genes, we performed a research GS analysis on the 76 prospectively collected cases without a detected causative variant by the clinical GS. The research analysis revealed two additional causative variants increasing the total diagnostic yield of our study to 23.4%. One of these variants was a maternally inherited variant in *ZFPM2* (case #50). The variant was filtered out in the clinical GS due to inheritance pattern. The mother was a healthy carrier of the variant, but since the ZFPM2‐related diaphragmatic hernia has known reduced penetrance,^27^ the variant was considered likely causative in the fetus with diaphragmatic hernia. The other variant detected by the research analysis was a de novo duplication in the *ATAD3* locus (case #74), which was assessed as likely benign in the primary analysis, as partial gene duplications where one of the breakpoints is located outside the gene do not usually cause gene disruption. When new knowledge regarding recurrent duplications in the *ATAD3* locus causing fusion genes with dominant‐negative effect became available,^28^ this duplication was considered causative in the fetus. The findings from the research analysis emphasize the limits of a trio genome analysis if inheritance pattern is used as a strict filtering criterium, especially with genes with reduced penetrance, and the importance of repeating genetic analyses after new information comes available.

To the best of our knowledge, our study is one of the few studies to present TAT for clinical GS in ongoing pregnancies. A key challenge in this setting is the considerable time pressure, particularly when results are needed before a defined legal limit for pregnancy termination. In previous studies, the TAT of GS in ongoing pregnancies has varied between approximately 2 and 4 weeks.^13^, ^14^, ^16^ At our department, the turnaround time depends on various factors, including the gestational age and whether the parents have decided to continue the pregnancy or not based on the ultrasound findings. Our study shows that we are able to provide results within a timeframe that supports clinicians and parents in decisions regarding pregnancy continuation, with a mean TAT of 12.5 days.

We also show that parents receiving a molecular diagnosis are more likely to terminate the pregnancy than parents with non‐diagnostic results from prenatal GS. However, we cannot rule out that the prenatal cases that received a molecular diagnosis had more severe symptoms than the cases that got non‐diagnostic results from prenatal GS. Even so, previous studies have also shown that parents opt for termination of pregnancy more often when they receive a pathological finding in the ES or GS analysis.^13^, ^16^, ^29^

In our study, we have six examples where a prenatally identified molecular diagnosis does not only affect the decision on possible pregnancy continuation but also has an impact on the management of the ongoing pregnancy and/or the postnatal period following the parents' decision to continue the pregnancy. For example, in case #5 with a prenatally verified pyruvate dehydrogenase E1‐alpha deficiency, a specific diet and additional treatment were started directly after birth against the otherwise possibly lethal condition. In case #86 with Noonan syndrome 1, the molecular diagnosis meant individual follow‐ups during the pregnancy as well as individualized postnatal management, including possible prophylactic treatment to prevent ventricular tachycardia. In case #214 with a causative variant in *SMARCC1* associated with congenital hydrocephalus, the prenatal diagnosis gave reassurance regarding the chosen delivery method and the follow‐up and avoidance of invasive shunt placement in the born child.

It should be emphasized that the prenatal GS result may also impact future pregnancies. First, the parents with a risk of recurrence due to the identified cause being an inherited disease can be offered preimplantation genetic testing or prenatal diagnosis in their next pregnancy. Second, albeit the recurrence risk is low if the cause was a de novo variant, prenatal diagnosis can still be offered. Third, even a negative result is informative since it substantially reduces the likelihood of a genetic cause with a high recurrence risk. The residual risk can be subject to discussion, but it can usually be considered relatively low.

## 5 CONCLUSION

This study was performed as a part of implementing GS in prenatal precision medicine of congenital malformations at the Department of Clinical Genetics and Genomics, Karolinska University Hospital, Stockholm, Sweden. In our cohort, which was prescreened for chromosome aberrations in most cases, trio GS detected a molecular diagnosis in 23.4% (51/218) of cases. Our experience of using GS in prenatally detected congenital malformations has taught us that GS can add clinically relevant information by explaining the underlying cause of the malformation, and often in a reasonable time before the parents need to decide on the continuation of the pregnancy. When GS is offered to a prenatal case according to the criteria presented in Table 3, we nowadays include a CNV analysis using GS data, which means that an additional CMA is not needed.

## AUTHOR CONTRIBUTIONS

Eini Westenius conceptualized the study, curated the data, performed the formal analyses, acquired funding, investigated, developed methodology, administrated the project, provided resources, managed software, validated the data, managed the visualization, wrote the first draft of the report, reviewed, and edited the report. Peter Conner conceptualized the study, investigated, developed methodology, administrated the project, provided resources, supervised the study, reviewed, and edited the report. Ellika Sahlin conceptualized the study, curated the data, investigated, developed methodology, provided resources, managed software, supervised the study, validated the data, reviewed, and edited the report. Hafdis T. Helgadottir curated the data, investigated, provided resources, managed software, validated the data, reviewed, and edited the report. Maria Pettersson curated the data, investigated, provided resources, managed software, validated the data, reviewed, and edited the report. Meeli Sirotkina investigated, provided resources, reviewed, and edited the report. Nikos Papadogiannakis investigated, provided resources, reviewed, and edited the report. Anna Lindstrand conceptualized the study, acquired funding, investigated, developed methodology, provided resources, supervised the study, reviewed and edited the report. Erik Iwarsson conceptualized the study, performed the formal analyses, acquired funding, investigated, developed methodology, administrated the project, provided resources, supervised the study, reviewed, and edited the report.

## FUNDING INFORMATION

This study was supported by grants from Stockholm County Council (AL, EI, EW, FoUI‐961392, FoUI‐961849 (combined residency and PhD training program)), Swedish Research Council (AL), the Swedish Brain Foundation (AL), Birgitta and Carl‐Axel Rydbeck Donation (EI), Karolinska Institutet (EI) and Sällsyntafonden (AL, EI, EW).

## CONFLICT OF INTEREST STATEMENT

The authors declare no conflicts of interest.

## ETHICS STATEMENT

The study was approved by the Swedish Ethical Review Authority (reference numbers 2021‐02985 with a date of approval 4/8/2021 and 2019‐04746 with a date of approval of 20/12/2019). A written consent from both parents was required for the research analysis. Due to the confidential nature of the data, the supporting data are not publicly available. Access to the data can be requested from the corresponding author.

## Supporting information


**Table S1.** Detailed information of the 218 cases.

**Table S2.** Summary of the causative variants detected by clinical genome sequencing. *ACMG* The American College of Medical Genetics and Genomics, *AD* Autosomal dominant, *AR* Autosomal recessive, *DEL* Deletion, *Dn* De novo, *Hemi* Hemizygous, *Het* Heterozygous, *Hom* Homozygous, *INDEL* Insertion and deletion, *Mat* Maternal, *MT* Mitochondrial inheritance, *OMIM* Online Mendelian Inheritance in Man, *Pat* Paternal, *SNV* Single nucleotide variant, *XLD* X‐linked dominant, *XLR* X‐linked recessive.

**Table S3.** Findings in the research analysis. *ACMG* The American College of Medical Genetics and Genomics, *AD* Autosomal dominant, *DUP* Duplication, *Dn* De novo, *Het* Heterozygous, *Mat* Maternal, *OMIM* Online Mendelian Inheritance in Man, *Pat* Paternal, *SNV* Single nucleotide variant.

**Table S4.** Summary of the potentially clinically relevant variants detected by clinical genome sequencing. *ACMG* The American College of Medical Genetics and Genomics, *AD* Autosomal dominant, *AR* Autosomal recessive, *Hemi* Hemizygous, *Het* Heterozygous, *Hom* Homozygous, *INDEL* Insertion and deletion, *Mat* Maternal, *OMIM* Online Mendelian Inheritance in Man, *Pat* Paternal, *SNV* Single nucleotide variant, *XLD* X‐linked dominant.

**Table S5.** Incidental findings. *ACMG* The American College of Medical Genetics and Genomics, *AD* Autosomal dominant, *AR* Autosomal recessive, *DEL* Deletion, *Dn* De novo, *DUP* Duplication, *Het* Heterozygous, *Hom* Homozygous, *INDEL* Insertion and deletion, *Mat* Maternal, *OMIM* Online Mendelian Inheritance in Man, *Pat* Paternal, *SNV* Single nucleotide variant, *XLR* X‐linked recessive.

## Data Availability

The data that support the findings of this study are available on request from the corresponding author. The data are not publicly available due to privacy or ethical restrictions.
