# Supplementary material for: Genome sequencing in prenatal precision medicine of congenital malformations: Clinical experience from a single center
Source: Acta Obstet Gynecol Scand. 2026 Nov 13:10.1111/aogs.70381. Online ahead of print. doi: 10.1111/aogs.70381 (PMC13768129; doi:10.1111/aogs.70381)
Supplement: Supplementary file 1 [file AOGS-9999-0-s001.docx]

Supporting Information Table S4.

| **Case** | **Type of variant** | **Gene** | **Transcript** | **Coding change** | **Zygosity** | **ACMG class** | **Diagnosis** | **OMIM#** | **Inheritance** | **Parental source** |
| --- | --- | --- | --- | --- | --- | --- | --- | --- | --- | --- |
| #25 | SNV/ INDEL | *NODAL* | NM_018055.5 | c.536_537del/p.(Glu179Val*98) | Het | 4 (PVS1_Strong, PM2_Moderate) | Heterotaxy, visceral, 5 | # 270100 | AD | Mat |
| #26 | SNV/ INDEL  SNV/ INDEL | *FLVCR2* | NM_017791.3 | c.434T>C / p.(Leu145Pro)  c.839C>T / p.(Pro280Leu) | Compound het | 3 (PM2_Moderate)  3 (PM2_Moderate, PM5_Supporting) | Fowler syndrome | # 225790 | AR | Mat Pat |
| #51 | SNV/ INDEL | *SLC9A3* | NM_004174.4 | c.920C>T/p.(Ser307Leu) | Hom | 3 (PM2_Moderate, PM3_Supporting) | Diarrhea 8, secretory sodium, congenital | # 616868 | AR | Mat/Pat |
| #102 | SNV/ INDEL | *IKBKG* | NM_0003639 | c.181C>T/p.(Leu61Phe) | Hemi | 3 (PM2_Moderate, PP1_Moderate) | Incontinentia pigmenti | # 308300 | XLD | Mat |
