# Supplementary material for: Genome sequencing in prenatal precision medicine of congenital malformations: Clinical experience from a single center
Source: Acta Obstet Gynecol Scand. 2026 Nov 13:10.1111/aogs.70381. Online ahead of print. doi: 10.1111/aogs.70381 (PMC13768129; doi:10.1111/aogs.70381)
Supplement: Supplementary file 2 [file AOGS-9999-0-s002.docx]

Supporting Information Table S2.

| **Case** | **Type of variant** | **Gene** | **Transcript** | **Coding change** | **Zygosity** | **ACMG class** | **Diagnosis** | **OMIM #** | **Inheritance** | **Parental source** |
| --- | --- | --- | --- | --- | --- | --- | --- | --- | --- | --- |
| #1 | SNV/ INDEL | *FBXO11* | NM_001190274 | c.2700dup/p.(Leu901Ilefs*4) | Het | 5 | Intellectual developmental disorder with dysmorphic facies and behavioral abnormalities | # 618089 | AD | Dn |
| #5 | SNV/ INDEL | *PDHA1* | NM_000284.3 | c.853_865dup/p.(Tyr289Serfs*12) | Het | 4 | Pyruvate dehydrogenase E1-alpha deficiency | # 312170 | XLD | Dn |
| #6 | SNV/ INDEL | *COL1A2* | NM_000089 | c.3089G>T/p.(Gly1030Val ) | Het | 4 | Osteogenesis imperfecta, type II | # 166210 | AD | Dn |
| #11 | SNV/ INDEL | *L1CAM* | NM_000425 | c.1681C>T/p.(Gln561*) | Hemi | 5 | Partial agenesis of corpus callosum | # 304100 | XLR | Dn |
| #12 | SNV/ INDEL | *FGFR3* | NM_000142 | c.1118A>G/p.(Tyr373Cys) | Het | 5 | Thanatophoric dysplasia type 1 | # 187600 | AD | Dn |
| #18 | SNV/ INDEL | *DHCR7* | NM_001360 | c.964-1G>C | Hom | 5 | Smith-Lemli-Opitz syndrome | # 270400 | AR | Mat/Pat |
| #21 | SNV/ INDEL | *ACTA1* | NM_001100 | c.814G>C/p.(Glu272Gln) | Het | 4 | Nemaline myopathy 3 | # 161800 | AD | Dn |
| #23 | SNV/ INDEL | *CDH2* | NM_001792.5 | c.2467C>T/p.(Arg823*) | Het | 5 | Agenesis of corpus callosum, cardiac, ocular and genital syndrome | # 618929 | AD | Dn |
| #28 | SNV/ INDEL | *FGFR3* | NM_000142.5 | c.1108G>T/p.(Gly370Cys) | Het | 5 | Thanatophoric dysplasia type 1 | # 187600 | AD | Dn |
| #29 | SNV/ INDEL  DEL | *FRAS1* | NM_025074 | c.6519del/p.(Lys2173Asnfs*12)  c.8753-370_8958+257del | Compound het | 5  5 | Fraser syndrome 1 | # 219000 | AR | Pat Mat |
| #33 | SNV/ INDEL | *SOX9* | NM_000326 | c.1320_1321insCACCA/p.(Asp441Hisfs*31) | Het | 5 | Campomelic dysplasia | # 114290 | AD | Dn |
| #41 | SNV/ INDEL | *OFD1* | NM_003611.3 | c.275_276del/p.(Ser92Cysfs*24) | Het | 5 | Orofaciodigital syndrome I | # 311200 | XLD | Dn |
| #49 | SNV/ INDEL | *EYA1** | NM_000503.6 | c.1319G>A/p.(Arg440Gln) | Het | 5 | Branchiootorenal syndrome 1 | # 113650 | AD | Pat |
| #55 | SNV/ INDEL | *L1CAM* | NM_000425 | c.1828+5G>A | Hemi | 4 | Partial agenesis of corpus callosum | # 304100 | XLR | Mat |
| #57 | SNV/ INDEL | *STAG2* | NM_006603.5 | c.1544_1547del/p.(Asp515Glyfs*6) | Het | 5 | Holoprosencephaly 13 | # 301043 | XLD | Dn |
| #60 | SNV/ INDEL | *NIPBL* | NM_133433.4 | c.5092G>T/p.(Glu1898*) | Het | 5 | Cornelia de Lange syndrome 1 | # 122470 | AD | Dn |
| #66 | SNV/ INDEL | *CHD7* | NM_017780.4 | c.7879C>T/p.(Arg2627*) | Het | 5 | CHARGE Syndrome | # 214800 | AD | Dn |
| #68 | SNV/ INDEL | *SMARCC1* | NM_003074.4 | c.1385+1G>C | Het | 4 | Hydrocephalus congenital, 5, susceptibility to | # 620241 | AD | Dn |
| #72 | SNV/ INDEL | *POMGNT1* | NM_017739 | c.1011dup/p.(Asp338*) | Hom | 5 | Muscular dystrophy-dystroglycanopathy (congenital with brain and eye anomalies), type A, 3 | # 253280 | AR | Dn  (Maternal uniparental isodisomy of chromosome 1) |
| #79 | SNV/ INDEL | *COL1A1*** | NM_000088 | c.1099del/p.(Gln367Argfs*174) | Het | 4 | Osteogenesis imperfecta, type I | # 166200 | AD | Mat |
| #82 | SNV/ INDEL | *RIT1* | NM_006912 | c.270G>A/p.(Met90Ile) | Het | 5 | Noonan syndrome 8 | # 615355 | AD | Dn |
| #84 | SNV/ INDEL | *SMC3* | NM_005445.4 | c.430-1G>C | Het | 5 | Cornelia de Lange syndrome 3 | # 610759 | AD | Dn |
| #86 | SNV/ INDEL | *PTPN11* | NM_001330437 | c.124A>G/p.(Thr42Ala) | Het | 5 | Noonan syndrome 1 | # 163950 | AD | Dn |
| #90 | SNV/ INDEL | *COL4A1* | NM_001845.6 | c.3592G>A/p.(Gly1198Arg) | Het | 5 | Brain small vessel disease with or without ocular anomalies | # 175780 | AD | Mat |
| #94 | SNV/ INDEL | *SOX9* | NM_000346 | c.1337_1343dup/p.(Ser449Argfs*131) | Het | 5 | Campomelic dysplasia with autosomal sex reversal | # 114290 | AD | Dn |
| #97 | SNV/ INDEL | *RPGRIP1L* | NM_015272.5 | c.2050C>T/p.(Gln684*) | Hom | 5 | Meckel syndrome 5 | # 611561 | AR | Mat/Pat |
| #105 | SNV/ INDEL  SNV/ INDEL | *GLE1* | NM_001003722 | c.1092dup/p.(Ala365Serfs*12)  c.1750C>T/p.(Arg584Trp) | Compound het | 4  3 | Lethal congenital contracture syndrome 1 | # 253310 | AR | Pat Mat |
| #115 | SNV/ INDEL | *SPECC1L* | NM_015330 | c.1220A>C/p.(Gln407Pro) | Het | 4 | Teebi hypertelorism syndrome 1 | # 145420 | AD | Mat |
| #116 | SNV/ INDEL | *SPECC1L* | NM_015330 | c.1220A>C/p.(Gln407Pro) | Het | 4 | Teebi hypertelorism syndrome 1 | # 145420 | AD | Mat |
| #118 | SNV/ INDEL | *TUBA1A* | NM_006009 | c.700A>T/p.(Ile234Phe) | Het | 4 | Lissencephaly 3 | # 611603 | AD | Dn |
| #156 | SNV/ INDEL | *COL5A1* | NM_000093.5 | c.3772G>T/p.(Gly1258*) | Het | 5 | Ehlers-Danlos syndrome, classic type, 1 | # 130000 | AD | Dn |
| #157 | SNV/ INDEL | *KMT2D* | NM_003482.4 | c.15142C>T/p.(Arg5048Cys) | Het | 5 | Kabuki syndrome 1 | # 147920 | AD | Dn |
| #159 | SNV/ INDEL | *ACTA1* | NM_001100 | c.341C>A/p.(Pro114His) | Het | 4 | Congenital myopathy 2C, severe infantile | # 620278 | AD | Dn |
| #160 | SNV/ INDEL | *PTPN11* | NM_002834.5 | c.181G>A/p.(Asp61Asn) | Het | 5 | Noonan syndrome 1 | # 163950 | AD | Dn |
| #163 | SNV/ INDEL | *EFTUD2* | NM_001258353 | c.1058+1G>A | Het | 4 | Mandibulofacial dysostosis, Guion-Almeida type | # 610536 | AD | Dn |
| #168 | SNV/ INDEL | *KMT2D* | NM_003482.4 | c.11233C>T/p.(Gln3745*) | Het | 5 | Kabuki syndrome 1 | # 147920 | AD | Dn |
| #169 | DEL  SNV/ INDEL | *CEP55* | NM_018131 | c.-1176_183+1013del/p.(Met1?) c.572G>A/p.(Arg191Gln) | Compound het | 4 4 | Multinucleated neurons, anhydramnios, renal dysplasia, cerebellar hypoplasia, and hydranencephaly | # 236500 | AR | Mat Pat |
| #177 | SNV/ INDEL | *ABAT* | NM_020686.6 | c.991G>A/p.(Gly331Arg) | Hom | 4 | GABA-transaminase deficiency | # 613163 | AR | Mat/Pat |
| #179 | SNV/ INDEL | *STAC3* | NM_145064.3 | c.855G>A/p.(Trp285*) | Hom | 4 | Congenital myopathy 13 | # 255995 | AR | Mat/Pat |
| #180 | SNV/ INDEL | *MT-ATP6 MT-ATP8* | NC_012920.1 | m.8528T>C | Homoplasmy | 4 | MT-ATP6 and MT-ATP8 related disease |  | MT | Mat (homoplasmy) |
| #184 | SNV/ INDEL | *SAMD9* | NM_017654.4 | c.2944C>T/p.(Arg982Cys) | Het | 5 | MIRAGE syndrome | # 617053 | AD | Dn |
| #188 | SNV/ INDEL | *GLE1* | NM_001003722 | c.433-10A>G | Hom | 4 | Lethal congenital contracture syndrome 1 | # 253310 | AR | Mat/Pat |
| #193 | SNV/ INDEL | *DNAH5* | NM_001369 | c.3396G>C | Hom | 4 | Ciliary dyskinesia, primary, 3, with or without situs inversus | # 608644 | AR | Mat/Pat |
| #194 | Chromosomal |  |  |  |  |  | Temple syndrome |  |  | Dn  (Maternal uniparental heterodisomy of chromosome 14) |
| #202 | SNV/ INDEL | *BRAF* | NM_004333.6 | c.1411G>T/p.(Val471Phe) | Het | 4 | Noonan syndrome 7 | # 613706 | AD | Dn |
| #204 | Chromosomal |  |  |  |  |  | Unbalanced translocation with a 730 kb terminal deletion of 6pterp25.3 and a 29 Mb terminal duplication of 12q23.3qter |  |  | Mother with t(6;12)(p25.3;q23.3) |
| #210 | SNV/ INDEL | *COL1A1* | NM_000088.4 | c.1436G>C/p.(Gly479Ala) | Het | 4 | Osteogenesis imperfecta, type II | # 166210 | AD | Dn |
| #214 | SNV/ INDEL | *SMARCC1* | NM_003074.4 | c.1723C>T/p.(Gln575*) | Het | 4 | Hydrocephalus, congenital, 5, susceptibility to | # 620241 | AD | Pat |
| #218 | SNV/ INDEL | *L1CAM* | NM_024003.3 | c.400+5G>T | Hemi | 4 | Hydrocephalus, congenital, X-linked | # 307000 | XLR | Mat |

*The variant in *EYA1* was inherited from the father, who turned out to have branchiootorenal syndrome with a hearing impairment and unspecified renal symptoms. However, the variant in *EYA1* was not considered to be able to explain the whole fetal phenotype, which included not only bilateral hydronephrosis but also tetralogy of Fallot.

**The variant in COL1A1 was inherited from the mother who had known osteogenesis imperfecta. However, the variant in COL1A1 was not considered to be able to explain the whole fetal phenotype, which included not only abnormal position of the legs but also abdominal symptoms.
