# Supplementary material for: Genome sequencing in prenatal precision medicine of congenital malformations: Clinical experience from a single center
Source: Acta Obstet Gynecol Scand. 2026 Nov 13:10.1111/aogs.70381. Online ahead of print. doi: 10.1111/aogs.70381 (PMC13768129; doi:10.1111/aogs.70381)
Supplement: Supplementary file 3 [file AOGS-9999-0-s003.docx]

Supporting Information Table S5.

| **Case** | **Type of variant** | **Gene** | **Transcript** | **Coding change** | **Zygosity** | **ACMG class** | **Diagnosis** | **OMIM#** | **Inheritance** | **Parental source** |
| --- | --- | --- | --- | --- | --- | --- | --- | --- | --- | --- |
| #36 | Chromosomal |  |  |  |  |  | Triple X |  |  |  |
| #37 | DUP |  |  |  |  |  | A 2.62 Mb interstitial duplication of 22q11.21 |  | AD | Mat |
| #48 | SNV/INDEL | *KCNQ1* | NM_000218 | c.573_577del/p.(Arg192Cysfs*91) | Het | 5 | Long QT syndrome 1 | # 192500 | AD | Mat |
| #61 | DEL | *DMD* | NM_004006.3 | c.6615-16420_7309+3025del | Het | 5 | Duchenne muscular dystrophy | # 310200 | XLR | Mat |
| #69 | Chromosomal |  |  |  |  |  | Turner syndrome |  |  |  |
| #122 | SNV/INDEL | *RASA1* | NM_002890 | c.2125C>T/p.(Arg709*) | Het | 5 | Capillary malformation-arteriovenous malformation 1 | # 608354 | AD | Pat |
| #123 | SNV/INDEL | *RASA1* | NM_002890 | c.2125C>T/p.(Arg709*) | Het | 5 | Capillary malformation-arteriovenous malformation 1 | # 608354 | AD | Pat |
| #129 | Chromosomal |  |  |  |  |  | Klinefelter syndrome |  |  |  |
| #145 | SNV/INDEL | *NFIB* | NM_001190737.2 | c.377A>C/p.(Lys126Thr) | Het | 4 | Macrocephaly, acquired, with impaired intellectual development | # 618286 | AD | Dn |
| #174 | Chromosomal |  |  |  |  |  | Unbalanced translocation with a 1,2 Mb terminal duplication of 8q24.3qter and a 1,3 Mb terminal deletion of 12p13.33pter |  |  | Mat |
| #179 | SNV/INDEL | *ATP7B* | NM_000053.4 | c.3182G>A/p.(Gly1061Glu) | Hom | 5 | Wilson disease | # 277900 | AR | Mat/Pat |
| #182 | SNV/INDEL | *FLCN* | NM_144997.7 | c.1285del/p.(His429Thrfs*39) | Het | 5 | Birt-Hogg-Dube syndrome | # 135150 | AD | Mat |
| #187 | SNV/INDEL | *CYP7B1* | NM_004820 | c.1456C>T/p.(Arg486Cys) | Hom | 5 | Spastic paraplegia 5A | # 270800 | AR | Mat/Pat |
| #192 | SNV/INDEL | *SCN5A* | NM_198056.3 | c.4434+5G>A | Het | 4 | Brugada syndrome 1 | # 601144 | AD | Mat |
| #193 | SNV/INDEL | *EYS* | NM_001142800.2 | c.749-1G>C | Hom | 5 | Retinitis pigmentosa 25 | # 602772 | AR | Mat/Pat |
| #208 | DUP |  |  |  |  |  | A 543 kb duplication of 16p11.2 |  | AD | Pat |
| #209 | DUP |  |  |  |  |  | A 543 kb duplication of 16p11.2 |  | AD | Pat |
