# Supplementary material for: Genome sequencing in prenatal precision medicine of congenital malformations: Clinical experience from a single center
Source: Acta Obstet Gynecol Scand. 2026 Nov 13:10.1111/aogs.70381. Online ahead of print. doi: 10.1111/aogs.70381 (PMC13768129; doi:10.1111/aogs.70381)
Supplement: Supplementary file 4 [file AOGS-9999-0-s004.docx]

Supporting Information Table S3.

| **Case** | **Type of variant** | **Gene** | **Transcript** | **Coding change** | **Zygosity** | **ACMG class** | **Diagnosis** | **OMIM #** | **Inheritance** | **Parental source** |
| --- | --- | --- | --- | --- | --- | --- | --- | --- | --- | --- |
| #50 | SNV/ INDEL | *ZFPM2* | NM_012082.4 | c.591del/p.(Phe198Leufs*15) | Het | 4 | Diaphragmatic hernia 3 | # 610187 | AD | Mat |
| #74 | DUP |  |  |  |  |  | A 69 kb duplication of 1p36.33 |  | AD | Dn |
